# World-Wide FINGERS: An International Network of Linked Multidomain Trials

**DOI:** 10.1093/geroni/igab046.259

**Published:** 2021-12-17

**Authors:** Francesca Mangialasche, Alina Solomon, Tiia Ngandu, Miia Kivipelto

**Affiliations:** 1 Division of Clinical Geriatrics, Stockholm, Stockholms Lan, Sweden; 2 Karolinska Institutet, Stockholm, Stockholms Lan, Sweden; 3 Finnish Institute for Health and Wefare, Public Health Promotoin Unit, Uusimaa, Finland

## Abstract

Risk reduction and prevention of dementia in older adults is a growing research area. In the Finnish Geriatric Intervention Study to Prevent Cognitive Impairment and Disability (FINGER randomized controlled trial) a 2-year multidomain intervention -dietary counseling, exercise, cognitive training, vascular and metabolic risk monitoring- improved cognition in older adults from the general population who had increased dementia risk. The intervention was associated also with improvement of other clinical outcomes (e.g., multimorbidity, functional status). The FINGER model is being adapted and tested in different populations and settings through the World-Wide FINGERS, the first global network of multidomain prevention trials, including over thirty countries. The network goal is to identify effective and feasible solution for dementia risk reduction across the spectrum of cognitive decline - from at-risk asymptomatic states to early-symptomatic stages. Through the World-Wide FINGERS-SARS-CoV-2 initiative, the network aims to assess the effects of the COVID-19 pandemic in older adults.

